# Supplementary material for: Retrograde mitochondrial transport regulates mitochondrial biogenesis in zebrafish neurons
Source: bioRxiv. 2025 Oct 1:2025.09.29.679307. Preprint. [Version 1] doi: 10.1101/2025.09.29.679307 (PMC12621953; doi:10.1101/2025.09.29.679307)
Supplement: Supplement 1 [file media-1.pdf]

Supplemental Information

Table S1.

**Reagent or Resource**	**Source**	**Identifier**	**RRID**
<u>Antibodies</u>			
Chicken anti-GFP	Aves	GFP-1020	AB_10000240
Rabbit anti-SSBP1	ProteinTech	12212-1-AP	AB_2195320
Rabbit anti-TFAM	ProteinTech	22586-1-AP	AB_11182588
Sheep anti-DIG-AP	Roche	11093274910	AB_514497
<u>Drugs</u>			
Resveratrol	MilliporeSigma	554325	
AICAR	Sigma-Aldrich	A9978	
<u>Experimental models: organisms/strains</u>			
Zebrafish: AB	ZIRC	AB	ZDB-GENO-960809-7
Zebrafish: *mitfa* (*nacre*)	[110]	w2	ZDB-ALT-990423-22
Zebrafish: *actr10*	[34]	nl15	ZDB-FISH-170719-8
Zebrafish: *p150a*	[36]	y625	ZDB-ALT-200527-5
Zebrafish: *p150b*	[34]	nl16	ZDB-ALT-170628-4
Zebrafish: *nudc*	[43]	nl21	ZDB-ALT-190722-1
Zebrafish: *TgBAC(neurod:eGFP)*	[111]	nl1	ZDB-ALT-080701-1
Zebrafish: *Tg(5kbneurod1:pgc1α-p2a-mRFP)*	[48]	uwd9	ZDB-ALT-231026-11
Zebrafish: *Tg(5kbneurod:mRFP-actr10)*	[36]	nl22	ZDB-ALT-201006-6
Zebrafish: *Tg(5kbneurod:mito-mEos)*	[36]	y586	ZDB-ALT-180628-3
Zebrafish*: Tg(hsp70l:EGFP-en.sill)*	This paper	uwd12	ZDB-ALT-250520-12
<u>Recombinant DNA</u>			
5kbneurod1:mito-eGFP	[48]		

5kbneurod1:mRFP	[34]
5kbneurod1:mitotagRFP	[34]
5kbneurod1:eGFP	[48]
Hsp701:Drp1K38A-mRFP	[34]
5kbneurod1:cox8a-cox8a-halotag	This paper
5kbneurod1:kif1a-egfp-omp25 ["MitoTruck"]	This paper
5kbneurod1:esrra-p2a-mRFP	This paper
5kbneurod1:cox8a-cox8a-paGFP-p2a-mitoTagRFP	This paper
5kbneurod1:cox8a-cox8a-NAD-p2a-RFP	This paper

Table S1: List of reagents and experimental models

Table S2.

Primer	Sequence
<u>Genotyping</u>	
actr10 FP	5’- CTGTTTTCGGATGAACTGCCTG
actr10 RP	5’- ACTTACTTCGTGTAGGCCGC
nudc FP	5’- GACCAGAGGCAGAAGTCCAT
nudc RP	5’- TTTTTGGCAGCTTGTTTTGAAAG
p150a FP	5’- TAGTGTGCAGATCCAATATGGC
p150a RP	5’- ATTTCCCAGAGGCGAAGAGT
p150b FP	5’- TCGGCTTCTGCAGGAGAGAT
p150b RP	5’- CTGGGTCCCGGGATTGGAG
<u>*in situ* probes</u>	
atp5md FP	5’- AATCATGGGTGGACACGACG
atp5md RP	5’- AGCATGAAAATTGGTGCGGG
cox5ab FP	5’- GATTTCAGCGGCGGTTCTTC
cox5ab RP	5’- GAAGTCAAACTCGGTCCCGT
nrf1 FP	5’- ACACAAGTTGCCACAACAGC
nrf1 RP	5’- ACAGGTACTCCTTGGAGGGA
pgc1a FP	5’- CTCGCAACATGGACGAAAGC
pgc1a RP	5’- AATGGACACACTTTGCGCAG
pgc1b FP	5’- AAAAGCGGATCTCAGCACCA
pgc1b RP	5’- TTCGGCCATCCAGAGGATTG
polb FP	5’- AACTGCGCAAGCTGGAAAAG
polb RP	5’- GCCGTTGTGGATCATCTGGA
tfam FP	5’- GGGGCTAATCTGCTGGTCAA
tfam RP	5’- CGGCTTTTGCGGAGGAAAAA
tomm40 FP	5’- GGACATGGACCAACACCGAA
tomm40 RP	5’- GGGCCTAACCTATCAGGCTG
<u>DNA plasmid generation</u>	
<u>*5kbneurod1:kif1a-egfp-omp25* ["MitoTruck"]</u>	
kif1a FP	5’- CTATAGGGCGAATTGGGTACATGGCCGGGGCCTCTGTG
kif1a RP	5’- TGCTCACCATGGTAGGCCTAGGCGCGCC
kif1a GFP FP	5’- TAGGCCTACCATGGTGAGCAAGGGCGGG
kif1a GFP RP	5’- GGCCGTTCTGTCTAGAGACGGTCCGCTTGTAC
omp25 MLS FP	5’- CGTCTCTAGACAGAACGGCCCCACCAGC
omp25 MLS RP	5’- TATCAAGCTTATCGATACCGTTAAAATGGGCCCCGGGG

5kbneurod1:cox8a-cox8a-halotag

cox8a_1 FP	5’- CTATAGGGCGAATTGGGTACATGTCCGTCCTGACGCCG
cox8a_1 RP	5’- GGACGGACATTGGGTCCAACGAATGGATCTTG
cox8a_2 FP	5’- GTTGGACCCAATGTCCGTCCTGACGCCG
cox8a_2 RP	5’- TTTCGGATCCTGGGTCCAACGAATGGATCTTG
HaloTag FP	5’- GTTGGACCCAGGATCCGAAATCGGTACTG
HaloTag RP	5’- TATCAAGCTTATCGATACCGTTAACCGGAAATCTCCAG

hsp70l:EGFP-en.sill

Hsp70 GFP FP	5’- TCCGCAGCCCCCAAGCTTGGATGGTGAGCAAGGGCGAG
Hsp70 GFP RP	5’- GTTGGGATGGCTATAGGGCTGCAGAATCTAGAG
SILL FP	5’- AGCCCTATAGCCATCCCAACTCACTCAC
SILL RP	5’- TGGATCATCATCGATGGTACCTGACATTTTCCGGAACAG

5kbneurod1:esrra-p2a-mRFP

esrra FP	5’- CTATAGGGCGAATTGGGTACATGTCTTCCAGAGAACGAC
esrra RP	5’- CTTTGTACAAGGGTGAGTCCATCATGGC
p2a mRFP FP	5’- GGACTCACCCTTGTACAAAGTGGGGGGATC
p2a mRFP RP	5’- TATCAAGCTTATCGATACCGTTACTTGTACAAGGCGCC

5kbneurod1:cox8a-cox8a-NAD-p2a-RFP

mitoNAD FP	5’- CTATGGGCGAATTGGGTACATGCTGGCCACCCGCGTG
mitoNAD RP	5’- CTCCGGATCCACCTACACGTTGTGTCGGCG
RFP FP	5’- ACGTGTAGGTGGATCCGGAGCCACGAAC
RFP RP	5’- TATCAAGCTTATCGATACCGTTACTTGTACAAGGCGCCG

5kbneurod1:cox8a-cox8a-paGFP-p2a-mitoTagRFP

Cox8a1 FP	5’-CTATAGGGCGAATTGGGTACATGTCCGTCCTGACGCCA
Cox8a1 RP	5’-GGACGGACATCAACGAATGGATCTTGGCAC
Cox8aPAGFP FP	5’-CCATTCGTTGATGTCCGTCCTGACGCCG
Cox8aPAGFP RP	5’-CTCCGGATCCCTTGTACAGCTCGTCCATG
2amitoTagRFP FP	5’-GCTGTACAAGGGATCCGGAGCCACGAAC
2amitoTagRFP RP	5’- TATCAAGCTTATCGATACCGTTATAGTTTGTGCCCCAGTTTGC

Table S2: List of oligonucleotide primers

Table S3.

Gene	ENSEMBL ID	Probe set size, amplifier
atf3	ENSDART00000022060.8	16, B2
atp5md	ENSDART00000150789.3	5, B1
cox5ab	ENSDART00000172518.2	10, B1
cox6c	ENSDART00000056319.4	6, B1
cox7c	ENSDART00000165249.2	5, B2
cox8a	ENSDART00000138236.2	8, B1
efhd1	ENSDART00000063779.4	8, B1
ndufa5	ENSDART00000103293.4	7, B2
ndufb3	ENSDART00000144625.3	7, B2
pgc1a	ENSDART00000097710.6	20, B1
polb	ENSDART00000002764.9	20, B1
spry4	ENSDART00000099528.4	18, B2
tfam	ENSDART00000092009.6	20, B2
tomm40	ENSDART00000015302.7	18, B2
uqcrq	ENSDART00000180722.1	5, B2

Table S3: List of probe sets used for HCR RNA FISH

Movie S1.
Related to Figure S4G. Live imaging of pLL axon mitochondria labeled with MitoTagRFP for a wild type larva 3 dpf. Retrograde transport is directed left, anterograde right. The acquisition rate is 0.3 s/frame. Scale bar = 5 μm.

Movie S2.
Related to Figure S4G. Live imaging of pLL axon mitochondria labeled with MitoTagRFP for a *actr10* mutant larva 3 dpf. Retrograde transport is directed left, anterograde right. The acquisition rate is 0.3 s/frame. Scale bar = 5 μm.

Movie S3.
Related to Figure S4G. Live imaging of pLL axon mitochondria labeled with MitoTagRFP for a wild type larva 4 dpf. Retrograde transport is directed left, anterograde right. The acquisition rate is 0.3 s/frame. Scale bar = 5 μm.

Movie S4.
Related to Figure S4G. Live imaging of pLL axon mitochondria labeled with MitoTagRFP for a *actr10* mutant larva 4 dpf. Retrograde transport is directed left, anterograde right. The acquisition rate is 0.3 s/frame. Scale bar = 5 μm.

Movie S5.
Related to Figure 3D. Timelapse of a 4 dpf wild type pLL axon terminal after cell body photoconversion (strategy outlined in Fig. 3A). Magenta are new (cell body-derived) mitochondria. Axon terminal area is outlined. The cell body is positioned on the left. Arrow indicates the direction of new mitochondria entering the axon terminal. Scale bar = 5 μm

Movie S6.
Related to Figure 3D. Timelapse of a 4 dpf *actr10* pLL axon terminal after cell body photoconversion (strategy outlined in Fig. 3A). Magenta are new (cell body-derived) mitochondria. Axon terminal area is outlined. The cell body is positioned on the left. Arrow indicates the direction of new mitochondria entering the axon terminal. Scale bar = 5 μm

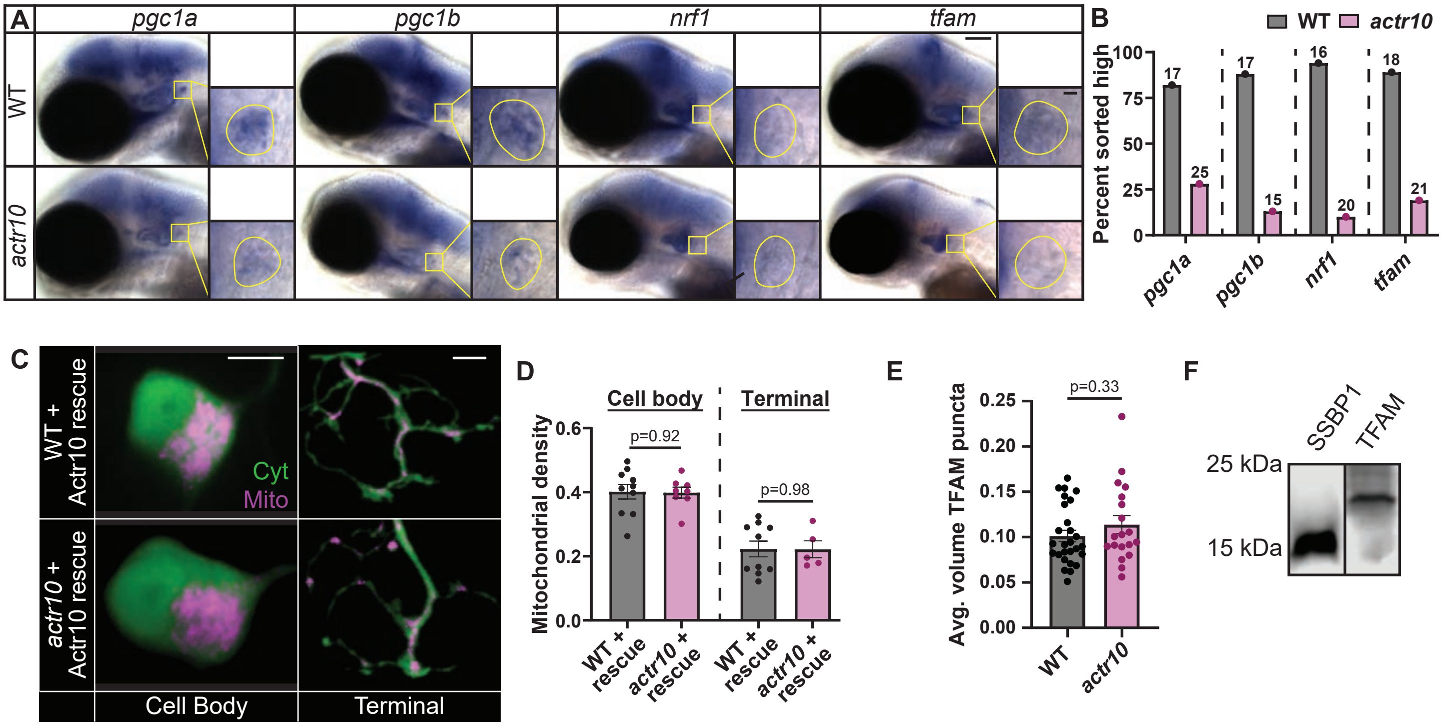

Figure S1: Mitochondrial biogenesis markers are reduced in *actr10* mutant neurons. (A) Digoxigenin-labeled in situ hybridization for *pgc1a, pgc1b, nrf1*, and *tfam* mRNA in 4 dpf zebrafish. Inset shows staining in the pLLg (outline). Scale bars: Head = 100 µm, inset = 10 µm. (B) Larvae were sorted into high or low mRNA expression in the pLLg or brain and genotyped post-sorting. Percentage of total wild type or *actr10* larvae sorted "high expression" is shown. Number of larvae per group indicated on graph. (C) 4 dpf pLL single cell body and axon terminal for wild type and *actr10* mutant larvae expressing Actr10 in neurons (+ Actr10 rescue; *Tg(neurod:mRFP-Actr10)*^{nl15}). Cytosolic area visualized with GFP (Cyt); mitochondria visualized with matrix-localized HaloTag (Mito, magenta). Scale bars = 5 µm. (D) Quantification of mitochondrial density (ANOVAs). (E) Average volume of TFAM puncta (Wilcoxon). (F) Anti-SSBP1 and Anti-TFAM western blots of wild type zebrafish larval protein extracts. All data are mean ± SEM. Data points represent individual larvae.

A Gene Set Enrichment Analysis of Gene Ontology

Description	Set Size	Enrichment Score (ES)	Normalized ES	p.adjust
mitochondrial protein-containing complex	210	-0.5326	-2.28005	4.54E-08
inner mitochondrial membrane protein complex	131	-0.56575	-2.29566	1.79E-06
mitochondrial inner membrane	275	-0.4498	-1.98734	8.85E-06
ribosome	178	-0.49632	-2.07555	1.30E-05
structural constituent of ribosome	150	-0.51605	-2.12838	3.11E-05
ribosomal subunit	144	-0.51706	-2.11537	4.92E-05
organelle inner membrane	292	-0.42636	-1.89981	4.92E-05
mitochondrial respiratory chain complex I	39	-0.73818	-2.40085	5.10E-05
NADH dehydrogenase complex	39	-0.73818	-2.40085	5.10E-05
respiratory chain complex I	39	-0.73818	-2.40085	5.10E-05

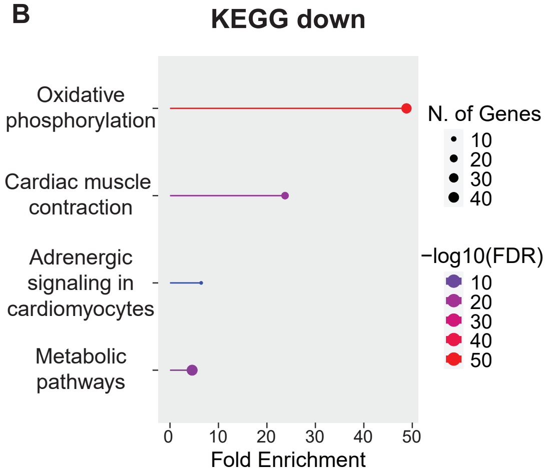

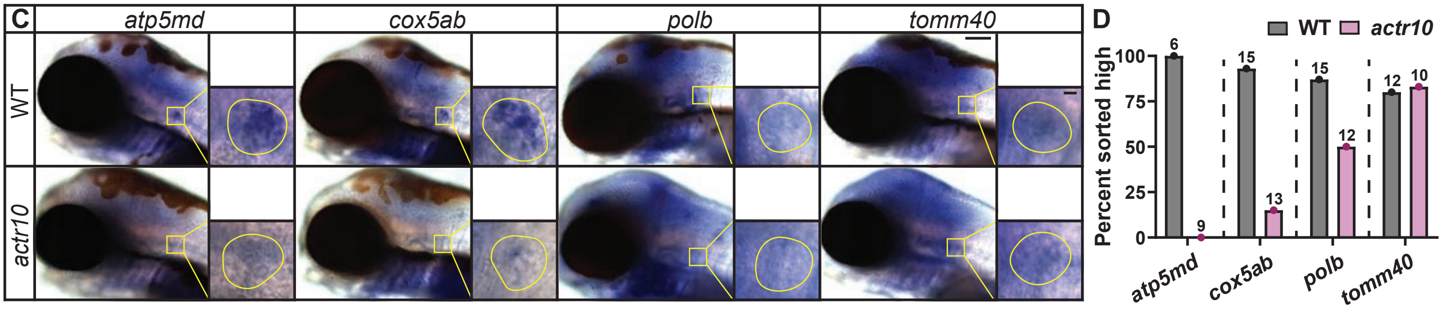

Figure S2: Mitochondrial gene transcription is impaired in *actr10* mutants. (A) Gene Set Enrichment Analysis results for the top 10 de-enriched gene ontology terms in *actr10* mutants relative to wild type. (B) Enrichment analysis of KEGG pathways for significantly downregulated genes in *actr10* mutants. (C) Digoxigenin-labeled in situ hybridization for *atp5md, cox5ab, polb,* and *tomm40* mRNA in 4 dpf larvae. Inset shows staining in the pLLg (outlined). Scale bars: Head = 100 µm, inset = 10 µm. (D) Larvae were sorted into high or low mRNA expression in the pLLg or brain and genotyped post-sorting. Percentage of total wild type or *actr10* larvae sorted "high expression" is shown. Number of larvae per group indicated on graph.

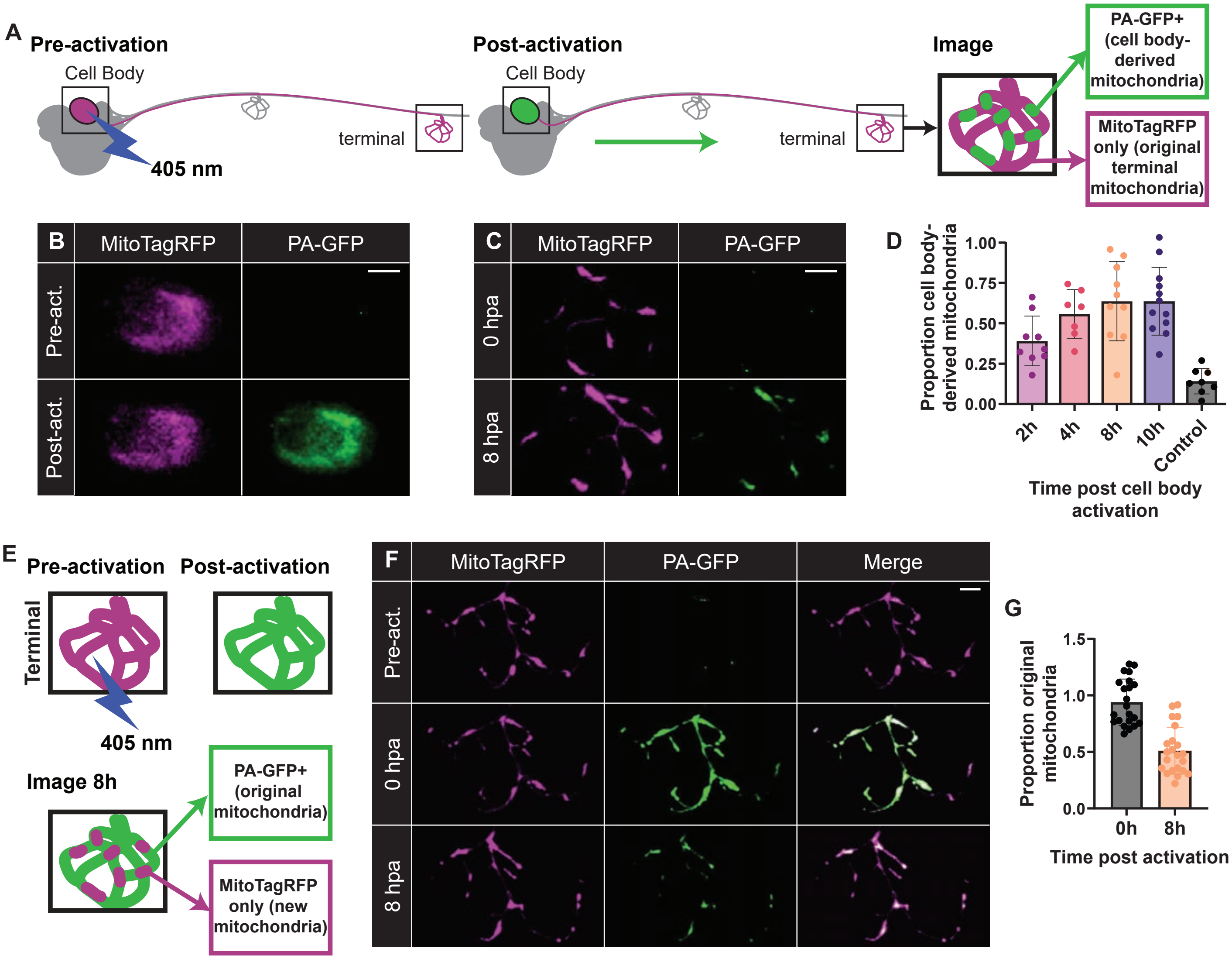

Figure S3: Cell body-derived mitochondria contribute significantly to the axon terminal. (A) pLL cell body activation strategy to quantify cell body-derived mitochondria that contribute to the axon terminal. A pLL cell body co-expressing MitoTagRFP and mitochondrial matrix-localized photoactivateable (PA) GFP was locally activated using 405 nm laser and the corresponding axon terminal was imaged at set time points post cell body activation (hpa). (B) A pLL cell body pre- and post-activation (act.). (C) A pLL axon terminal 0 hpa and 8 hpa. (D) Proportion of cell body derived (PA-GFP+) mitochondria in the axon terminal relative to all axon terminal mitochondria (MitoTagRFP+) at multiple time points post cell body activation. Control was an axon terminal imaged at 0h and 8h with no cell body activation. (E) Terminal photoactivation strategy to assess mitochondrial turnover. A pLL axon terminal co-expressing MitoTagRFP and mitochondrial matrix-localized PA-GFP was locally activated using 405 nm laser and imaged 8 hpa. (F) A pLL axon terminal pre-activation, immediately post-activation (0 hpa) and 8 hpa. (G) Proportion of original (PA-GFP+) mitochondria in the axon terminal relative to all mitochondria (MitoTagRFP+). Scale bars = 5 µm. All data are mean ± SEM; data points represent individual larvae.

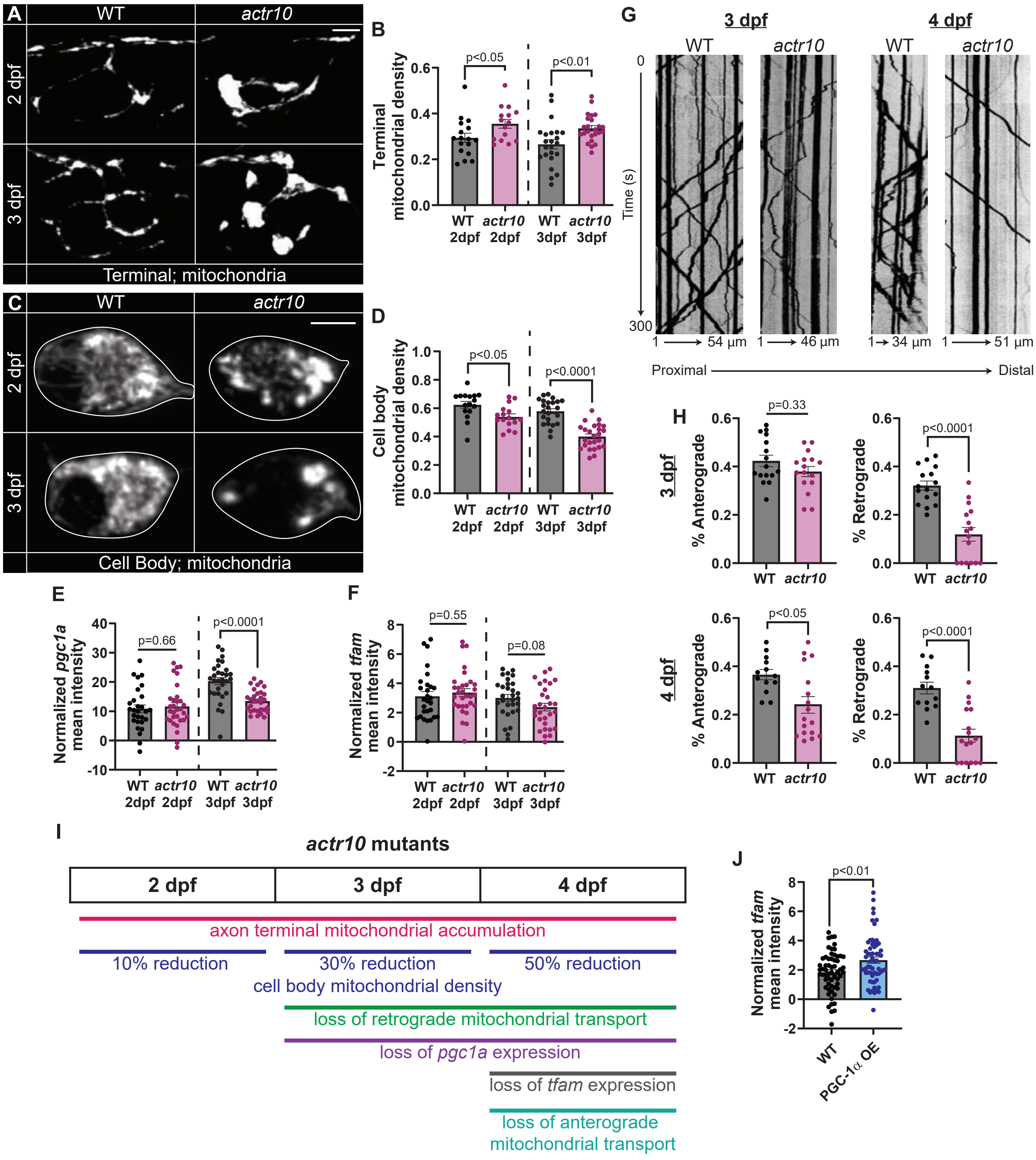

Figure S4: *actr10* mutants display progressive loss of mitochondrial biogenesis. (A) pLL axon terminals expressing mitoTagRFP in 2 and 3 dpf wild type or *actr10* mutant larvae. (B) Quantification of mitochondrial density (ANOVAs). (C) Representative images of single pLL cell bodies (white outline) expressing mitoTagRFP in 2 and 3 dpf wild type and *actr10* mutant larvae. (D) Quantification of mitochondrial density (ANOVAs). (E, F) Mean fluorescence intensity of *pgc1a* (E) and *tfam* (F) HCR RNA FISH normalized to background in the pLLg of 2 and 3 dpf wild type and *actr10* mutant larvae (ANOVAs). (G) Representative kymographs of mitochondrial transport in wild type and *actr10* pLL axons at 3 and 4 dpf. (H) Mitochondrial transport frequencies (Wilcoxons). Frequencies represent the percentage of mitochondria moving anterogradely or retrogradely out of total mitochondria in the region imaged. (I) Summary of changes to mitochondrial localization and transport relative to mitochondrial biogenesis measures over time in *actr10* mutants compared to wild type controls. (J) pLLg mean fluorescence intensity of *tfam* HCR RNA FISH normalized to background for wild type vs. PGC-1α OE (ANOVA). Scale bars = 5 µm. All data are mean ± SEM and data points represent individual larvae.

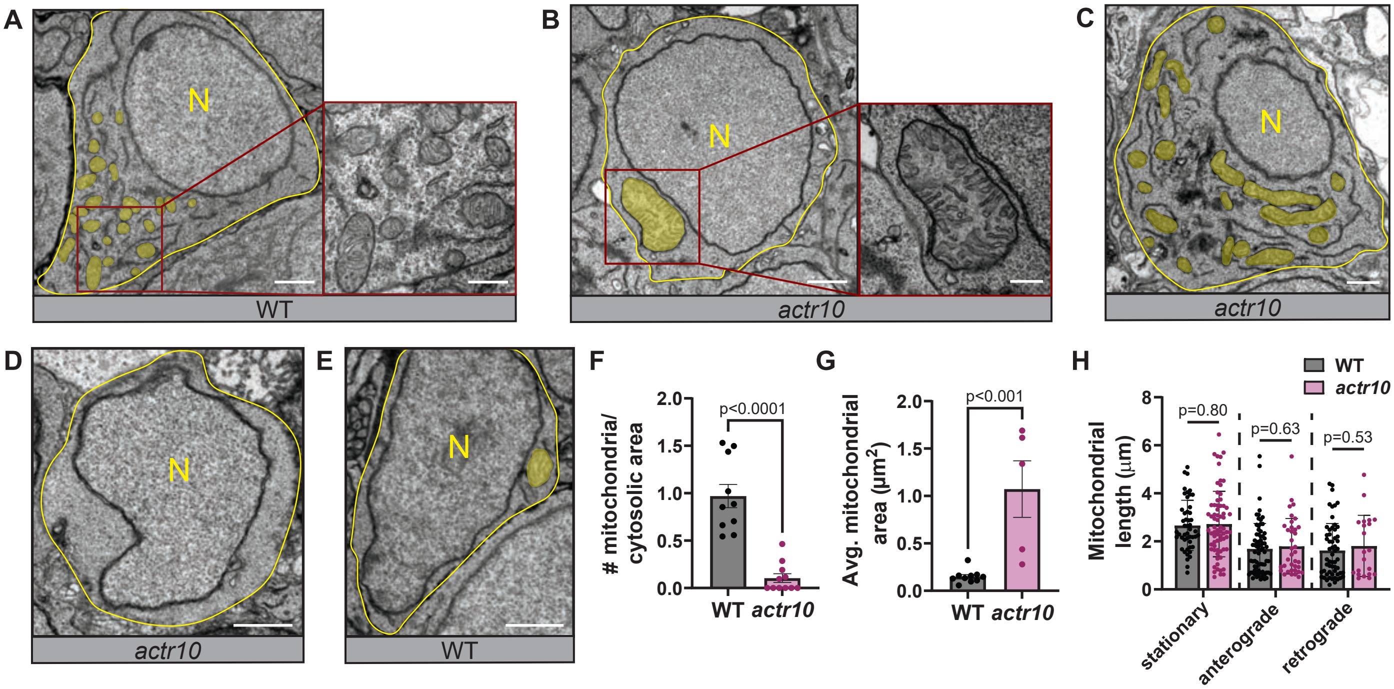

Figure S5: Mitochondrial structure in *actr10* mutants. (A-E) TEM images of pLL cell bodies. Cell area outlined in yellow, nucleus marked with "N," and mitochondria shaded in yellow. Scale bars: Full cell = 1 μm, insets = 400 nm. (F, G) Quantification of number of mitochondria relative to cytosolic area (cell area - nuclear area) and average mitochondrial area from TEM images (Wilcoxons). TEM data points represent individual cell bodies from one ganglion per genotype. (H) Length of stationary and motile mitochondria in wild type vs. *actr10* mutant pLL axons at 4 dpf. Data points represent all mitochondria measured from 10 wild type and 15 *actr10* mutant axons (ANOVAs). All data are mean ± SEM.

A TFEA.ChIP Transcription factor enrichment of downregulated genes

TF	Cell Type	Treatment	log2(OR)	log10(adj. p-value)	Euclidean Distance
ESR1	MCF-7	ethanol	2.8140	1.6742	6.2604
ZNF527	HEK293T	N/A	2.2369	1.2989	3.9344
ESRRG	BT-474	N/A	1.4826	3.3718	3.8196
MED12	MCF-7	CTL	2.0359	1.7679	3.5692
ESRRA	BT-474	N/A	1.3432	3.0862	3.4478
SOX2	NPC	R1159Q	2.0844	0.9761	3.3848
EZH2	myotube	N/A	1.2964	2.9988	3.3336
TCF3	Jurkat	N/A	2.0275	0.7168	3.1594
TP53	MOLM-13	M237I Daunorubicin	1.9877	0.9321	3.1091
TP53	MOLM-13	Y220C Daunorubicin	1.9877	0.9321	3.1091
EZH2	A-673	N/A	1.2510	2.7131	3.0440
EZH2	HUVEC	VEGF 12h	1.2808	2.6658	3.0250
EP300	HCT-116	Nutlin3a	1.8991	1.2757	3.0133
REST	neuroblastoma	SKNMM	1.7623	1.7851	2.9849
YAP1	HUCCT1	N/A	1.9569	0.6898	2.9636
EZH2	LNCaP	N/A	1.1427	2.6407	2.9039
SUZ12	HEK293T	PCGF1352fl	1.3262	2.4649	2.8892
TP53	MCF-7	NUT	1.8560	1.0619	2.8271
ZNF774	HEK293	N/A	1.8896	0.6657	2.7861
REST	NCI-H295R	SF1	1.6389	1.7685	2.7564
EZH2	hepatocyte	N/A	1.0598	2.3325	2.5724
ZNF274	GM08714	N/A	1.8178	0.3966	2.5564
AR	DU145	ARG56W	1.7708	0.8293	2.5510
EZH2	fibroblast	dermal	1.0443	2.1829	2.4277
EZH2	VCaP	DHAT 2H	1.2190	2.0300	2.4257
EZH2	THP-1	N/A	0.9693	2.2256	2.4230
ESRRA	BT-474	AICAR	0.9514	2.2304	2.4180
JARID2	UtE-iPS-7	N/A	1.0166	2.1829	2.4108
EPAS1	HUVEC	16h 1%O2	1.7337	0.6140	2.4054
NOTCH1_NICD	REC-1	GSI-mock-washout	1.7337	0.6140	2.4054

B Transcription factor target enrichment of downregulated genes

Gene Set	Set Size	Enrichment Ratio	p-value	FDR
TGACCTY_V$ERR1_Q2	755	3.0122	1.99E-07	6.55E-05
TGACCTTG_V$SF1_Q6	190	5.9847	2.14E-07	6.55E-06
TGACATY_UNKNOWN	475	2.578	0.00094	0.1656
GCANCTGNY_V$MYOD_Q6	655	2.2702	0.00109	0.1656
V$AMEF2_Q6	192	3.6446	0.00154	0.1656
V$NKX61_01	156	3.9249	0.00200	0.1656
V$SF1_Q6	202	3.4641	0.00212	0.1656
YTATTTTNR_V$MEF2_02	518	2.364	0.00216	0.1656
V$MMEF2_Q6	208	3.3642	0.00254	0.17355
V$OCT1_05	176	3.4789	0.00393	0.22473

Figure S6: Transcription factor enrichment analysis of genes downregulated in *actr10* mutants identifies Estrogen related receptors. (A) Transcription factor enrichment analysis for genes significantly downregulated in *actr10* mutants using TFEA.ChIP. Top 30 transcription factors (TF) based on euclidean distance from log2(Odds Ratio)/log10(adjusted p-value) to 0 are listed. (B) Transcription factor target enrichment analysis showing consensus motifs in the promoters of significantly downregulated genes.
